# Gas-Transport and the Dielectric Properties of Metathesis Polymer from the Ester of *exo*-5-Norbornenecarboxylic Acid and 1,1′-Bi-2-naphthol

**DOI:** 10.3390/polym14132697

**Published:** 2022-06-30

**Authors:** Ivan V. Nazarov, Danila S. Bakhtin, Ilya V. Gorlov, Konstantin V. Potapov, Ilya L. Borisov, Ivan V. Lounev, Igor S. Makarov, Alexey V. Volkov, Eugene Sh. Finkelshtein, Maxim V. Bermeshev

**Affiliations:** 1 A.V. Topchiev Institute of Petrochemical Synthesis, Russian Academy of Sciences, 29 Leninsky Prospekt, 119991 Moscow, Russia; vanyanaz@ya.ru (I.V.N.); db2@ips.ac.ru (D.S.B.); giv@ips.ac.ru (I.V.G.); boril@ips.ac.ru (I.L.B.); makarov@ips.ac.ru (I.S.M.); avolkov@ips.ac.ru (A.V.V.); fin314@gmail.com (E.S.F.); 2 Faculty of Fundamental Physical and Chemical Engineering, The Moscow State University, 1 Leninskie Gory, 119991 Moscow, Russia; 3 N.D. Zelinsky Institute of Organic Chemistry, Russian Academy of Sciences, 47 Leninsky Prospect, 119991 Moscow, Russia; kospotapov@yandex.ru; 4 Institute of Physics, Kazan Federal University, 18 Kremlyovskaya Street, 420008 Kazan, Russia; lounev75@mail.ru

**Keywords:** norbornene, metathesis polymerization, membrane gas separation, dielectric permittivity, polymeric dielectric materials

## Abstract

Polymers from norbornenes are of interest for applications in opto- and microelectronic (low dielectric materials, photoresists, OLEDs). Norbornenes with ester motifs are among the most readily available norbornene derivatives. However, little is known about dielectric properties and the gas-transport of polynorbornenes from such monomers. Herein, we synthesized a new metathesis polymer from *exo*-5-norbornenecarboxylic acid and 1,1′-bi-2-naphthol. The designed monomer was obtained via a two-step procedure in a good yield. This norbornene derivative with a rigid and a bulky binaphthyl group was successfully polymerized over the 1st generation Grubbs catalyst, affording high-molecular-weight products (M_w_ ≤ 1.5·10^6^) in yields of 94–98%. The polymer is amorphous and glassy (T_g_ = 161 °C), and it shows good thermal stability. Unlike most, polyNBi is a classic low-permeable glassy polymer. The selectivity of polyNBi was higher than that of polyNB. Being less permeable than polyNB, polyNBi unexpectedly showed a lower value of dielectric permittivity (2.7 for polyNBi vs. 5.0 for polyNB). Therefore, the molecular design of polynorbornenes has great potential to obtain polymers with desired properties in a wide range of required characteristics. Further tuning of the gas separation efficiency can be achieved by attaching an appropriate substituent to the ester and aryl group.

## 1. Introduction

Strained cycloalkenes have attracted much attention as building blocks in organic chemistry and as monomers in the synthesis of various polymers. There are several significant benefits of cycloalkenes in the synthesis of polymers. First, there is their increased reactivity in polymerization processes due to the high strain energy of a carbocycle. This feature allows involvement in polymerization monomers, bearing two, three, or four substituents, which is often impossible for a polymerization of most olefins. Second, there are at least two ways for polymerization of cycloalkenes (ring-opening metathesis polymerization and vinyl (addition) polymerization [1,2,3,4,5]), which give polymers with different structure and properties. Third, owing to well-developed organic tools, such as cycloaddition reactions [6,7], a huge number of cycloalkenes can be prepared, and this provides an opportunity to perform systematic structure–property investigations.

Norbornene derivatives are among the most available and highly reactive cycloolefins, which are synthesized from petrochemical and bio-feedstock. Polymers from norbornenes are of interest for applications in opto- and microelectronic (low dielectric materials, photoresists, OLEDs etc.) [8,9,10,11], membrane separation processes (gas separations [12], fuel cells [13], and pervaporation [14]), as well as materials for adhesives [15], sensors [16], and catalyst supports [5,17]. Interestingly, similar strategies are used in the development of gas separation polymeric membrane materials and materials with low permittivity [12,18]. For example, the highest gas permeable polymers are rigid polymers, containing bulky side-chains (e.g., polyacetylenes [19], PIMs [20], polynorbornenes [21]). This is due to a large free volume content in these polymers. The same polymers usually also exhibit low dielectric permittivity [22,23,24,25,26]. Therefore, it is a promising strategy to study designed polymers for both applications (as a gas-separation material and as a polymeric dielectric). The analysis of the literature data has shown that there is a lack of information about gas-transport and the dielectric properties of polynorbornenes with ester moieties in side-groups. To the best of our knowledge [27], there is not a study of gas permeability of such type of polymers. As to the dielectric properties, there is also little known about the properties of polymers from norbornenes with ester motifs. The dielectric permittivity of vinyl-addition poly(norbornene dicarboxylic acid dialkyl ester)s and poly(norbornene dimethyl dicarboxylate)s were evaluated in works [28,29]. The polymers exhibited dielectric permittivity up to 3.5 at 1 MHz. Recently, Opris et al. published on synthesis and the dielectric properties of metathesis polynorbornenes with ester-fragments in side-chains [30]. These polynorbornenes showed moderate room temperature dielectric permittivity and high dielectric relaxation strength.

In this work, we have designed and synthesized a new polymer based on motifs widely used in organic chemistry and polymer science, *exo*-5-norbornenecarboxylic acid and (S)-1,1′-bi-2-naphthol. The gas-transport and dielectric properties of this polymer have been studied to elucidate the effect of the incorporation of ester groups in side-chains on the characteristics of the polymer. The results obtained are considered with data of TGA, DSC, and WAXD analysis, and they are compared with earlier described data for the related polymers.

## 2. Experimental Section

### 2.1. Materials

The 1-st generation Grubbs catalyst, oxalyl chloride, methyl iodide, triethylamine, (S)-1,1′-bi-2-naphthol and solvents from Sigma-Aldrich were used in the current investigation. *Exo*-5-norbornene-2-carboxylic acid was synthesized according to the procedure published in the literature [31]. 1,2-Dichloroethane (DCE) was dried over CaH_2_ and distilled under argon. CH_2_Cl_2_ was dried over CaH_2_ and distilled in air.

The syntheses were carried out under argon using standard Schlenk techniques. Metathesis polymerization was carried out in the glovebox by M.Braun Incorporated, Stratham, NH, USA.

### 2.2. Methods of Monomer’s and Polymer’s Characterization

NMR-spectra were recorded on a Bruker Ascend 400 spectrometer at 400.1 MHz (^1^H), 100.6 MHz (^13^C) and on a Bruker Avance NEO spectrometer at 300 MHz (^1^H), 75 MHz (^13^C). Chemical shifts (δ) were reported in parts per million (ppm) relative to the reference (residual CHCl_3_ signal) for ^1^H and ^13^C NMR spectra. Each sample was dissolved in CDCl_3_ up to a concentration of 10%.

High-resolution mass spectra were obtained using micrOTOF II by Bruker Daltonic (Bremen, Germany) with simultaneous electrospray ionization (Skyline v2.1 by MacCoss Lab Software, Seattle, WA, USA)

Film mechanical testing was carried out on A I1140M-5-01-1 universal tensile testing machine (Tochpribor-KB, Ivanovo, Russia) and the ASTM D638 method was used for film mechanical testing. Dog-bone tensile specimens (ASTM standard D1708-96, 22 × 5 mm) were prepared by punching the films from a stainless steel die. The data is presented as the mean  ±  standard deviation (SD) and median with interquartile range. One-way ANOVA followed by Dunnett’s multiple comparisons test was performed using Sigma Stat 3.5 (Systat Software, San Jose, CA, USA). Values of *p* < 0.05 were considered statistically significant.

Gel-permeation chromatography (GPC) analysis of the polymers was performed on Agilent 1280 Infinity II system (Agilent GPC/SEC Software, version A.02.01 Build 9.34851 [12] by Agilent Technologies, Santa Clara, CA, USA) with a differential refractometer (THF as the eluent, flow rate 0.3 mL/min). Molecular mass and polydispersity were calculated by standard procedure relative to monodispersed polystyrene standards.

Calorimetric measurements were conducted using a “Mettler” TA-4000 differential scanning calorimeter (Giesen, Germany) at a heating rate of 20 °C/min under argon. TGA measurements were carried out on “TGA/DSC 1” (Mettler Toledo, Polaris Parkway, OH, USA) in argon and in air at the heating rate of 10 °C/min from 30 to 1000 °C.

Wide-angle X-ray diffraction (WAXD) data were obtained using a two-coordinate AXS detector (Bruker, Bremen, Germany) and Cu Kα emission (wavelength of 0.154 nm).

Specific rotation was measured using KRÜSS P3000 polarimeter by KRÜSS, Hamburg, Germany in CHCl_3_ or THF (HPLC grade).

Circular dichroism (CD) spectra of the polymers were measured in chloroform at the concentration of 1 mg/mL. Spectra were recorded with spectropolarimeter J810 (Jasco, Japan) in the 220–450 nm range (50 nm/min, 1 nm slit width) in 0.1 cm path-length quartz cells with a detachable window (Hellma, Germany). The baseline spectrum was recorded from pure chloroform.

Preparation the electrodes for dielectric measurements. The electrodes were polished by a special polishing paste (GOI) with particles size 1–7 μm until mirror shine. Further, the electrodes were rinsed by acetone 2–3 times, distillated water 2–3 times, and dried by air. After that, the film was placed behind the electrodes into measuring head ZGS with special mechanism for plane-parallel pressing.

Dielectric measurements were preformed in the frequency range of 0.01 Hz–10 MHz over the temperature interval of –100 to +200 °C using a BDS Concept-80 dielectric spectrometer (Novocontrol, Montabaur, Germany) at the Laboratory of Dielectric Spectroscopy, FCCU, Institute of Physics, Kazan Federal University. Discs with a diameter of 22 mm were cut from the polymer’s films. For the measurement of dielectric permittivity, the relative measurement error was below 3%. Automatic temperature control was achieved by employing a QUATRO cryosystem with a temperature stability better than 0.5 °C. The cell for samples for experiments with dielectrics consisted of a capacitor with parallel plates containing electrodes with actual diameters of 20 mm, the distance between the electrodes depended on the thickness of the sample. The thickness of the sample was measured with a micrometer with an accuracy of no worse than 0.005 mm. The experimental temperature protocol was as follows: each sample was placed in the measurement cell; starting from room temperature, the samples were cooled down to –100 °C at a rate of 10 °C/min. The samples were then measured in the temperature interval of –100 to +200 °C with a step of 5 °C.

### 2.3. Film Preparation 

The polymer films for gas permeation, dielectric and mechanical measurements were prepared by casting from a 5 wt% chloroform solution of the polymer. The solution was poured into a steel cylinder with a diameter of 7 cm and a stretched cellophane bottom. The solvent was allowed to evaporate slowly at room temperature to yield the desired polymer films. After the formation of the films, the cellophane was detached, and the films were dried under a vacuum at room temperature to a constant weight. A thermal treatment was not applied. The thickness of the films formed was in the range of 80–100 µm. The properties of the obtained membranes were measured immediately after evacuation. The time of sample investigation was 2 days.

### 2.4. Measurements of Gas-Transport Properties

Gas permeability and diffusion coefficients of gases were determined according to the Daynes–Barrer technique using a precise unit “Helmholtz-Zentrum Geesthacht” mounted with a pressure sensor Baratron (MKS Instruments, accuracy 10^−7^ bar) at 30 °C. The permeability coefficient is given in Barrer. The sorption coefficient was calculated as the permeability coefficient ratio to the diffusion coefficient.

### 2.5. Synthesis of the Monomer

(S)-2′-Methoxy-[1,1′-binaphthalen]-2-ol was prepared according to the published procedure [32]. K_2_CO_3_ (579 mg, 4.19 mmol) was added to the solution of (S)-1,1′-bi-2-naphthol (1.0 g, 3.49 mmol) in 20 mL of dry acetone. The mixture was stirred at rt for 1h. Then, methyl iodide (496 mg, 3.49 mmol) was added and the resulting suspension was refluxed for 48 h. The mixture was filtered and the solid washed with acetone. The filtrate was concentrated in vacuo and the residue purified by column chromatography (CH_2_Cl_2_/hexane 2:1) to give (S)-2′-methoxy-[1,1′-binaphthalen]-2-ol as a colorless solid (770 mg, 73%).


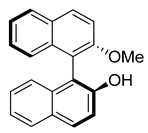


(S)-2′-Methoxy-[1,1′-binaphthalen]-2-ol

^1^H NMR (300 MHz, CDCl_3_, δ, ppm): 8.03 (d, *J* = 9.1 Hz, 1H, ArH), 7.93–7.81 (m, 3H, ArH), 7.46 (d, *J* = 9.1 Hz, 1H, ArH), 7.40–7.31 (m, 1H, ArH), 7.34 (d, *J* = 8.7 Hz, 1H, ArH), 7.31–7.28 (m, 1H, ArH), 7.27–7.25 (m, 1H, ArH), 7.21–7.14 (m, 2H, ArH), 7.07–7.01 (m, 1H, ArH), 4.91 (s, 1H, OH), 3.78 (s, 3H, CH_3_) (in Supplementary Materials). 


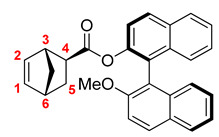


(S)-2′-Methoxy-[1,1′-binaphthalen]-2-yl bicyclo [2.2.1]hept-5-ene-2-carboxylate

Oxalyl chloride (4.23 g, 33.29 mmol) was added to the suspension of *exo*-5-norbornene-2-carboxylic acid (920 mg, 6.66 mmol) in 25 mL of dry CH_2_Cl_2_, containing 3 drops of DMF. The mixture was stirred at rt for 8 h. The resulting solution was evaporated to dryness. The residue was dissolved in 10 mL of dry CH_2_Cl_2_ and added dropwise to the ice-cooled mixture of triethylamine (1.68 g, 16.65 mmol) and (S)-2′-methoxy-[1,1′-binaphthalen]-2-ol (2.0 g, 6.66 mmol) in 50 mL of dry CH_2_Cl_2_. After the reaction mixture was stirred for 16 h at room temperature, it was quenched with water. The mixture was extracted with CH_2_Cl_2_. The organic layer was washed with brine, dried over anhydrous Na_2_SO_4_, filtered, and concentrated. The crude product was purified by column chromatography (hexane/EtOAc = 40:1 to 10:1) to afford the desired product (2.36 g, 84%) as a colorless oil (the mixture of diastereomers, dr = 1:1). [α]_D_^21^ = −17.7^o^ in THF (C = 1). HRMS (ESI): calcd. for C_29_H_24_O_3_ [M+nNH_4_]^+^438.2064, found 438.2065.

#### 2.5.1. Diastereomer A

^1^H NMR (300 MHz, CDCl_3_, δ, ppm): 8.03–7.88 (m, 1H, Ar*H*), 7.82 (t, *J* = 8.3 Hz, 1H, Ar*H*), 7.50–7.35 (m, 1H, Ar*H*), 7.34–7.07 (m, 2H, Ar*H*), 5.94 (m, 1H, *H*C = CH), 5.82 (m, 1H, HC = C*H*), 3.76 (s, 3H, OC*H*_3_), 2.57 (s, 1H, C*H*), 2.10–1.97 (m, 1H, CH-CO_2_Me), 2.04–2.00 (m, 1H, C*H*), 1.52–1.41 (m, 1H, CH_2_(5, a)), 1.05–0.89 (m, 1H), 0.78–0.64 (m, 1H,), 0.44 (d, *J* = 8.8 Hz, 1H).

^13^C NMR (75 MHz, CDCl_3_, δ, ppm): 174.01 (C = O), 154.96 (Ar-OMe), 146.97 (Ar-CO_2_R), 138.07 (C = C), 135.40 (C = C), 133.76 (Ar), 131.80 (Ar), 129.97 (Ar), 129.05(Ar), 128.14 (Ar), 127.67 (Ar), 126.58 (Ar), 126.44 (Ar), 126.10 (Ar), 125.52 (Ar), 125.40 (Ar), 125.36 (Ar), 123.72 (Ar), 122.08 (Ar), 118.02 (Ar), 113.68 (Ar), 56.87 (OCH_3_), 46.30 (C(4)), 45.47 (C(7)), 43.05, 41.43, 29.37.

#### 2.5.2. Diastereomer B

^1^H NMR (300 MHz, CDCl_3_, δ, ppm): 8.03–7.88 (m, 1H, Ar*H*), 7.82 (t, *J* = 8.3 Hz, 1H, Ar*H*), 7.50–7.35 (m, 1H, Ar*H*), 7.34–7.07 (m, 2H, Ar*H*), 5.94 (m, 1H, HC = CH), 5.82 (m, 1H, HC = CH), 3.72 (s, 3H, OCH_3_), 2.57 (s, 1H), 2.27 (s, 1H), 2.10–1.97 (m, 1H, CH-CO_2_Me), 1.25–1.14 (m, 1H), 1.05–0.89 (m, 1H), 1.01–0.93 (m, 1H), 0.78–0.64 (m, 1H).

^13^C NMR (75 MHz, CDCl_3_, δ, ppm): 174.07 (C = O), 155.00 (Ar-OMe), 146.93 (Ar-CO_2_R), 137.89 (C = C), 135.40 (C = C), 133.74 (Ar), 131.80 (Ar), 129.97 (Ar), 129.05 (Ar), 128.14 (Ar), 127.72 (Ar), 126.63 (Ar), 126.44 (Ar), 126.10 (Ar), 126.06 (Ar), 125.45 (Ar), 125.36 (Ar), 122.13 (Ar), 122.08 (Ar), 118.02 (Ar), 113.68 (Ar), 56.71 (OCH_3_), 45.81 (C(4)), 46.03 (C(7)), 42.96, 41.39, 29.70 (C(5)).

### 2.6. Metathesis Polymerization

The monomer (0.97 g, 2.3 mmol) was dissolved in 44 mL of absolute dichloroethane, and the solution of the 1-st generation Grubbs catalyst 0.97 mL (0.58·10^−3^ mmol, 3.36·10^−3^ M) was added to the monomer solution at stirring. The stirring continued for 30 min. The polymerization was terminated by the addition of ethyl vinyl ether followed by stirring for 5 min. Then, the polymer solution was precipitated by methanol containing the inhibitor (2,2′-methylenebis(6-*tert*-butyl-4-methylphenol)). The polymer was separated, washed by several portions of methanol, and dried in a vacuum. The polymer was twice reprecipitated by methanol from the chloroform solution and dried in a vacuum to a constant weight. The polymer yield was 0.91 g (94%). *M*_w_ = 1.5·10^6^, *M*_w_/*M*_n_ = 3.4. [α]_D_^21^ = −28^o^ in CHCl_3_ (C = 1).

^1^H NMR (400 MHz, CDCl_3,_ δ, ppm): 8.15–6.80 (m, 12H, ArH), 5.51–4.52 (m, 2H, HC = CH), 3.93–0.62 (m, 10H).

^13^C NMR (100 MHz, CDCl_3,_ δ, ppm): 173.63–173.32 (C = O), 154.99–154.95, 146.84–146.80, 133.8–122.05, 117.76–117.71, 113.66–113.60, 56.72–56.67, 50.10–49.70, 46.83–46.72, 41.95–41.76, 36.71–35.63.

## 3. Results and Discussion

### 3.1. Synthesis and Physico-Chemical Properties of the Polymer

**Figure 1:**
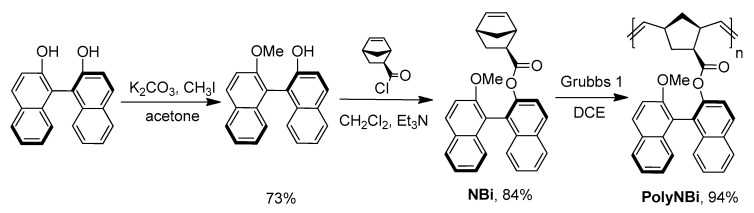
The synthesis and metathesis polymerization of the ester of *exo*-5-norbornenecarboxylic acid and 1,1′-bi-2-naphthol.

The target monomer was readily prepared in a good yield by the usage of (S)-1,1′-bi-2-naphthol and *exo*-5-norbornenecarboxylic acid as starting compounds (Figure 1). (S)-1,1′-Bi-2-naphthol contains two reactive OH-groups, and preliminary one of these OH-groups was converted in the ether group via the Williamson reaction, with iodomethane. The structure and the purity of the synthesized new norbornene derivative were confirmed by NMR-spectroscopy and high-resolution mass-spectrometry.

The obtained monomer was successfully involved in ring-opening metathesis polymerization in the presence of the 1-st generation Grubbs catalyst to produce high-molecular weight polymers in 94–98% yields (Figure 1 and Table 1).

The prepared metathesis polymer was soluble in common organic solvents (DCE, chloroform, and toluene). High-molecular weight polyNBi exhibited good film-forming and mechanical properties. The values of Young’s modulus (E), tensile strength (σ), and elongation at break (ε) were consistent with the properties for conventional glassy polymers (E = 2.4 ± 0.2 GPa, σ = 36 ± 6 MPa, and ε = 5 ± 2%).

**Figure 2:**
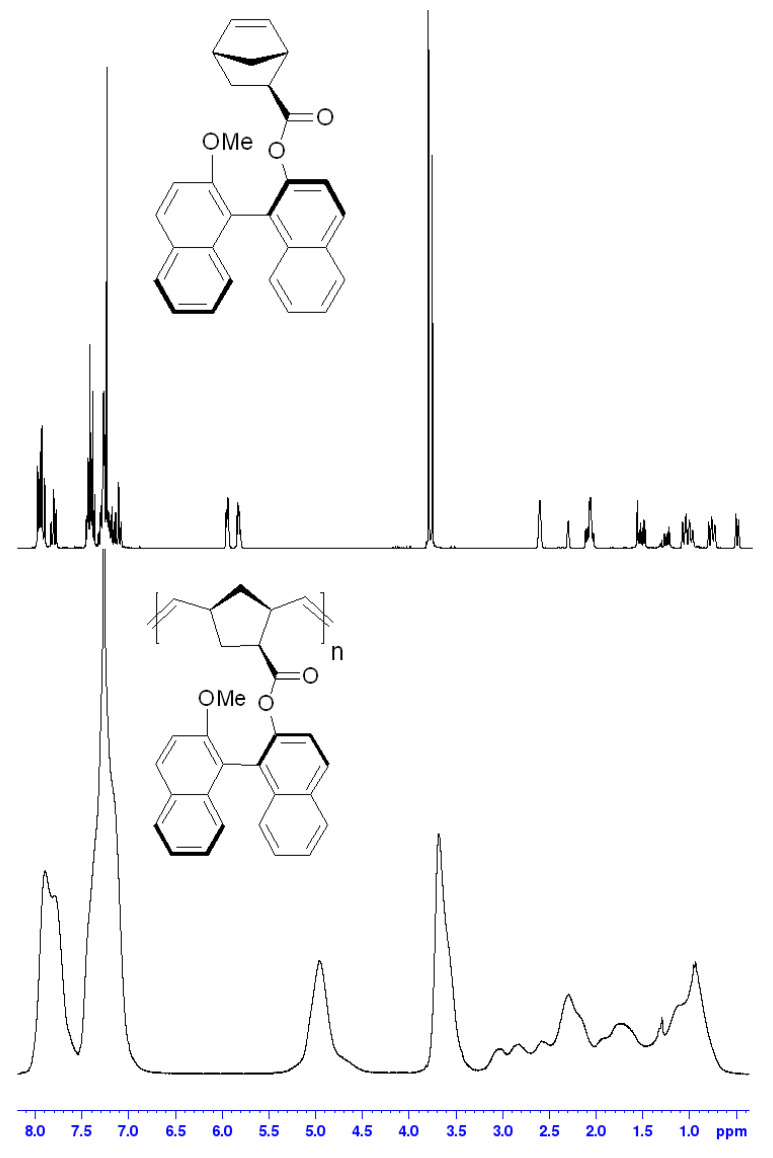
^1^H NMR spectra of the monomer and metathesis polymer based on the ester of *exo*-5-norbornenecarboxylic acid and 1,1′-bi-2-naphthol (CDCl_3_).

**Figure 3:**
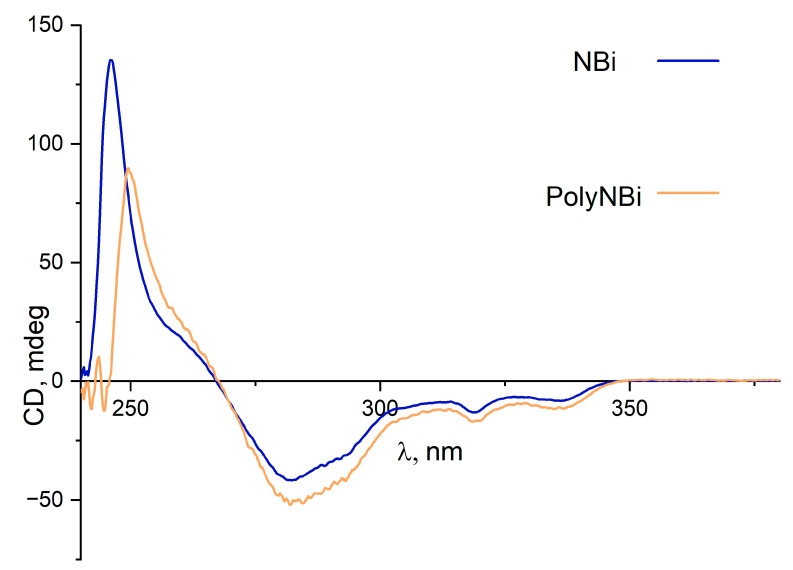
Circular dichroism spectra of the monomer and metathesis polymer based on the ester of *exo*-5-norbornenecarboxylic acid and 1,1′-bi-2-naphthol in CHCl_3_ at 25 °C (the concentration of both samples is 1 mg/mL).

The structure and the purity of the metathesis polymer based on the ester of *exo*-5-norbornenecarboxylic acid and 1,1′-bi-2-naphthol were analyzed with help of NMR spectroscopy (Figure 2). Since we used one of the isomers of 1,1′-bi-2-naphthol (*S*-isomer), the resulting monomer and the corresponding metathesis polymer were optically active compounds. The specific optical rotation for both compounds was different from zero (Table 1, experimental section). Circular dichroism (CD) spectra of the monomer and the synthesized metathesis polymer are very similar, showing a negative Cotton effect, with crossover at 260–270 nm (Figure 3).

**Figure 4:**
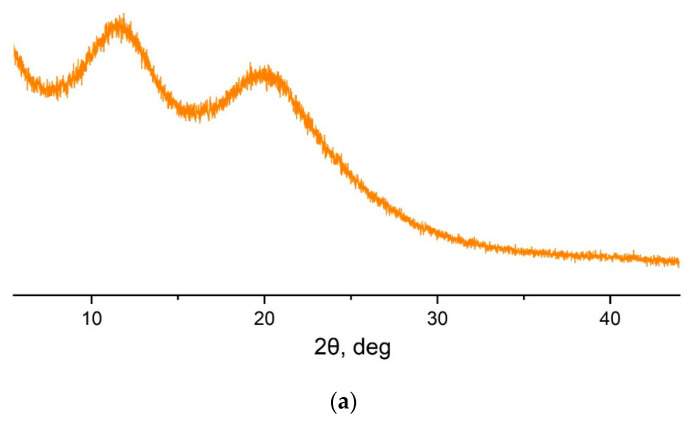

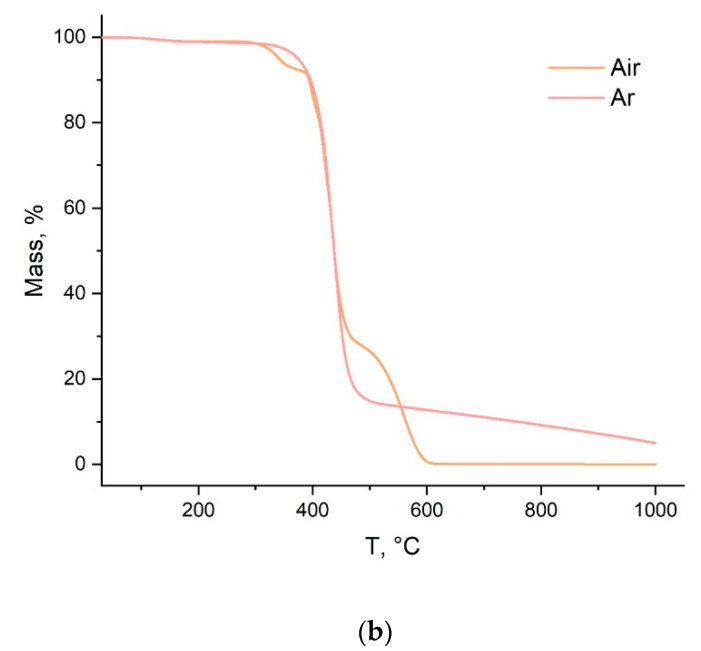
Wide-angle X-ray diffraction (WAXD) pattern (**a**) and TGA curves (**b**) for polyNBi.

The synthesized polyNBi is an amorphous polymer. Its WAXD pattern is represented by two broad peaks (Figure 4a). Most amorphous glassy polymers display one broad peak in their WAXD patterns. Therefore, the presence of two peaks for polyNBi in its WAXD pattern is not typical of an amorphous glassy polymer. This evidences that it is above the intermediate order. The intermediate level of order is believed due to intramolecular interactions and, probably, the helical structure of polyNBi. Rezac et al. earlier showed for various polynorbornenes that the peaks at high 2θ in WAXD patterns are attributed to intrasegmental interactions and the peaks at low 2θ to intersegmental interactions [33,34]; d-Spacings for polyNBi are larger than that of for the related unsubstituted metathesis polynorbornene (polyNB, Table 2). Therefore, the type of WAXD pattern and the position of peaks indicate that polyNBi has a more complex architecture of the packing of polymer chains.


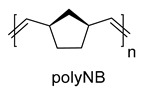


The structure of metathesis polynorbornene (polyNB).

According to DSC analysis, polyNBi is glassy, and its glass transition temperature is above 100 °C (Table 1). The value of T_g_ for polyNBi is much higher than that of the related polymer without substituents, polyNB (41 °C [35]). PolyNBi exhibited high thermal stability under argon and in air (the loss of 5 mass.% was above 300 °C, Figure 4b).

### 3.2. Gas-Transport Properties

The gas-transport characteristics of polyNBi were studied by the Daynes–Barrer method. Permeability (P) and diffusivity (D) coefficients of various gases (He, H_2_, N_2_, O_2_, CO_2_, CH_4_, and C_2_H_6_) in polyNBi were experimentally measured (Table 3). At the same time, solubility coefficients (S) were calculated by the usage of the well-known ratio *S* = *P*/*D*. The *p* values for polyNBi decrease in the following order: P(CO_2_) > P(He) > P(H_2_) > P(O_2_) > P(CH_4_) > P(N_2_) > P(C_2_H_6_). The change in diffusivity coefficients for polyNBi corresponds to the kinetic diameter of gas molecules: D(O_2_) > D(N_2_) > D(CO_2_) > D(CH_4_). At the same time, the highest solubility coefficients of gases in polyNBi were for the most easily condensable gases—CO_2_ and CH_4_—correspondingly: S(CO_2_) > S(CH_4_) > S(O_2_) > S(N_2_) (Table 3).

Interestingly, it has been earlier shown that the introduction of bulky groups in side-chains of polynorbornenes usually leads to an increase in gas permeability [27]. Herein, despite the presence of bulky side-substituents, the gas permeability of polyNBi is lower than that of unsubstituted metathesis polynorbornene, polyNB (Table 3). This is probably the result of the presence of polar ester motifs, which ensure a denser packing of polymer chains due to the dipole–dipole interaction. An additional explanation of the obtained result can be a low segmental mobility of chains in polyNBi due to π- π-stacking of aryl groups. This also explains why, despite the large interplanar distances in polyNBi compared to polyNB, the former has lower gas permeability. Due to the low segmental mobility in polyNBi, gas molecules hardly penetrate between the free volume elements, which leads to low diffusion coefficients. Therefore, the gas-transport properties of polyNBi are closer not to polynorbornenes but to polyarylenes, such as polysulfone (Table 3 and Table 4) in which there are strong π–π interactions.

Another interesting feature is the diffusivity-controlled permeability of hydrocarbons through polyNBi. Usually, such type polymers with hydrocarbonic or organosilicon-side groups possess solubility-controlled permeability of hydrocarbons [38,39]. Nevertheless, although there are large carbocyclic groups in side-chains of polyNBi, permeability of C_2_H_6_ was noticeably lower than CH_4_ permeability. Thus, this study shows that the influence of the ester and aryl fragment on gas permeability is stronger than the influence of the hydrocarbon group in the ester moiety.

**Figure 5:**
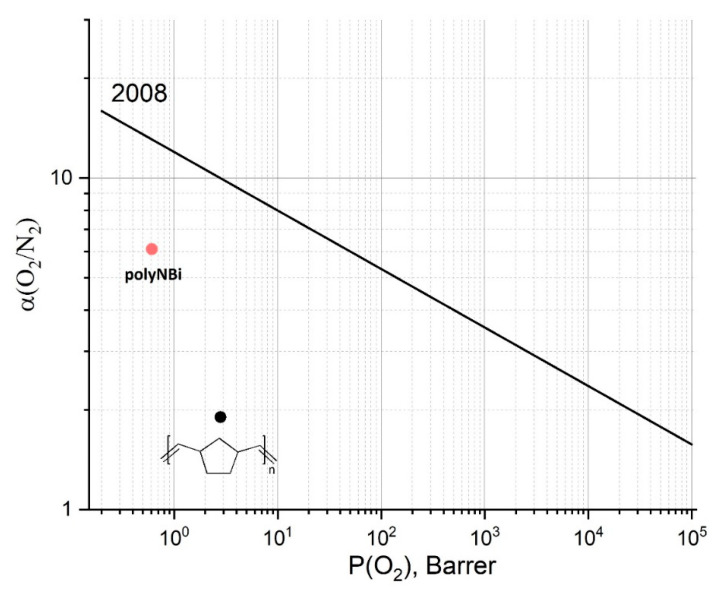
Robeson plot of O_2_/N_2_ separation performance for polyNB (black) and polyNBi (red).

Permeability selectivities for polyNBi were estimated as the ratio of permeability (P) coefficients of gas A to gas B (α = P_A_/P_B_). The corresponding values of α for various pairs of gases are given in Table 4. The highest values of selectivities of separations were found for pairs of gases that differ significantly in their sizes (H_2_/N_2_ and H_2_/CH_4_). Interestingly, these selectivities are much higher than those of polyNB (3–6 times higher). At the same time, for pairs of gases with similar molecular sizes, the selectivity of polyNBi was only 1.4–2.8 times higher than that of polyNB. The high selectivity for the O_2_/N_2_ and the He/CH_4_ gas pairs for polyNBi also draws attention. The value of O_2_/N_2_ selectivity is one of the highest for all studied polynorbornenes [27], and the data of polyNBi lie closer to the 2008 Robeson upper bound for the O_2_/N_2_ gas pair compared to the data of more permeable polyNB (Figure 5).

Based on the equation P = D·S, we factorized permeability selectivity of separation in two components—diffusivity selectivity and solubility selectivity:

α(A/B) = P(A)/P(B) = (D(A)/D(B))·(S(A)/S(B)) = α_D_·α_S_

where α_D_ and α_S_ are the contributions of diffusivity selectivity and solubility selectivity, respectively. The values of α_D_ and α_S_ for polyNBi are presented in Table 4. It can be seen that the main contribution to permeability selectivities for H_2_/N_2_ and H_2_/CH_4_ pairs is made by diffusivity selectivity. Thus, the selectivity of polyNBi is closer to those of classical amorphous polymers such as PSF than to polynorbornenes. While the contribution of solubility selectivity to permeability selectivity for other pairs of gases is comparable or greater than that of α_D_. This is especially pronounced for CO_2_-containing gas pairs because of the increased solubility coefficients of carbon dioxide in polyNBi (Table 3). Thus, unlike most, polyNBi is a classic low-permeable glassy polymer. This is unique to polynorbornenes with large side substituents, which tend to be highly permeable glasses. Therefore, the molecular design of polynorbornenes makes it possible to obtain polymers with desired properties in a wide range of required characteristics. Further tuning of the gas separation efficiency can be achieved by attaching an appropriate substituent to the ester and aryl group.

### 3.3. Dielectric Properties

**Figure 6:**
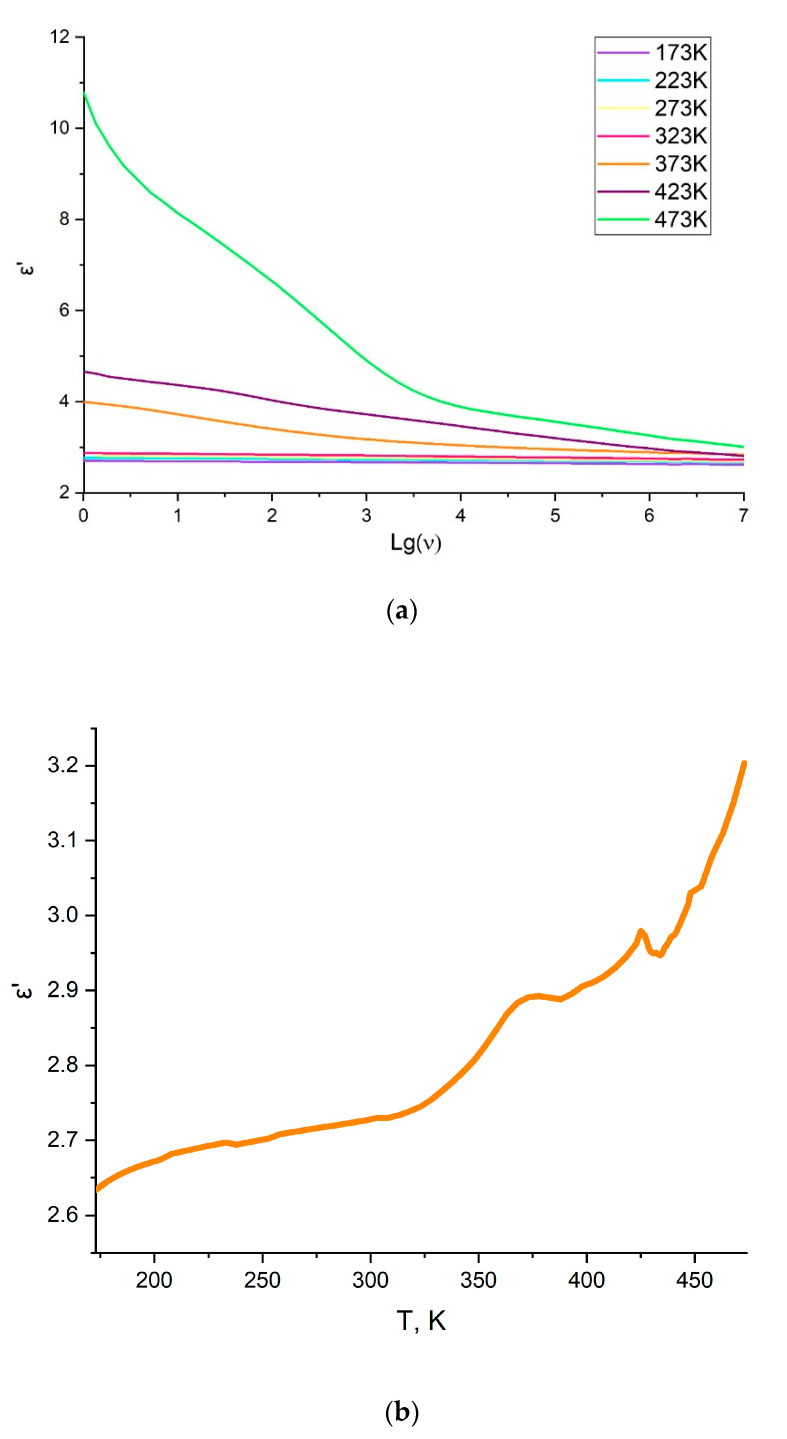
The frequency dependences (**a**) and the temperature dependence ((**b**), at 1 MHz) of ε′ for polyNBi.

Similar approaches are often used to develop gas separation membrane materials and low dielectric materials [12,18]. Therefore, it was also interesting to evaluate dielectric properties of the synthesized new polymer, polyNBi. The study of dielectric properties of polyNBi was conducted in the frequency range of 10^−2^ Hz–1·10^7^ Hz and in the temperature range of 173 to 473 K. The dependences of the dielectric constant versus frequency and temperature are shown in Figure 6 and the values of the dielectric properties of polyNBi (the dielectric constant (ε′), dielectric loss (ε″), and the loss tangent (tanδ = ε″/ε′, since the conductivity (σ) of polyNBi is very low; δ is a loss angle)) are summarized in Table 5. PolyNBi exhibited a significantly lower value of ε′ in comparison with the related polymer without side-substituents, polyNB. This is an interesting and unexpected result. Usually polymers with a larger free volume and a higher gas permeability have lower ε′ values [22]. However, due to the rigidity of the polymer chain and the π–π-stacking of aryl groups, the mobility of the main and the side chains in polyNBi is significantly hindered. In turn, the lower mobility of the structural fragments of the polymer should lead to an increase in the dielectric relaxation time and, consequently, to lower values of the permittivity.


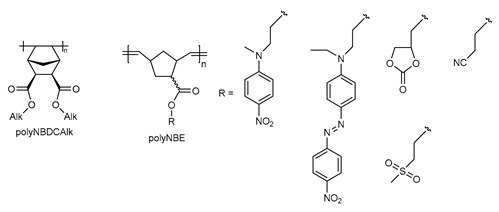


The structure of some addition and metathesis polynorbornene with ester motifs in side chains.

It is worth comparing the properties of polyNBi with the properties of related polymers based on norbornenecarboxylic acid and its derivatives. To the best of our knowledge, the first study of such type metathesis polymers was published only recently [30]. The corresponding polynorbornenes were prepared from esters of norbornenecarboxylic acid with fragments, bearing additional polar groups (polyNBE, Table 5). PolyNBE showed dielectric permittivity in the range of 3.3–8.9 at a room temperature that was higher than that of polyNBi. Since high dielectric relaxation strength (up to 19) was found for polyNBE, these polymers were considered as electrets, and they can be used in thermal sensors. Dielectric properties of vinyl-addition polymers from the dialkyl esters of norbornenedicarboxylic acid (polyNBDCAlk, Table 5) were described by Yoon et al. [29]. The dielectric constants for these vinyl-addition polynorbornenes ranged from 3.5 to 3.0, depending on the length of alkyl-group in ester motifs. Therefore, despite more rigidity of main chains and a less dense packing of vinyl-addition polymers, polyNBDCAlks showed higher values of ε′ compared to the studied polyNBi. This clearly evidences that the nature of alkyl group in the ester motif of side-chains can have a strong influence on dielectric permittivity through various kinds of interactions (dipole–dipole, π–π, etc.) and the incorporation of ester motifs with aryl groups in side-chains of polymers is a promising tool to decrease ε′.

**Figure 7:**
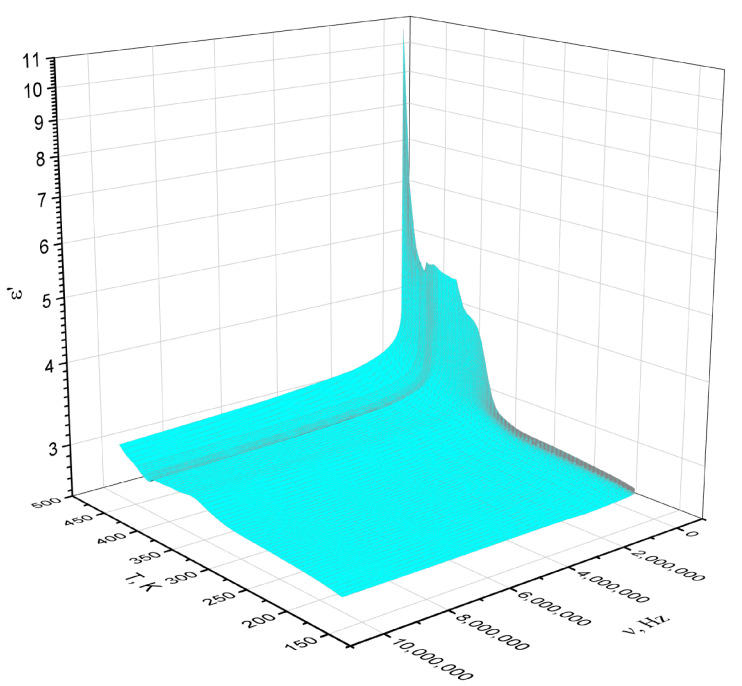
3D plot of dielectric permittivity versus temperature and frequency for polyNBi.

In Figure 7, the dependence of ε′ for polyNBi on the temperature and the frequency of the electric field is presented as a 3D plot. It can be seen that the temperature and frequency influence on the dielectric permittivity is weakly expressed at temperatures below T_g_ and at low frequencies. Therefore, this range of temperatures and frequencies is the most suitable for a potential application of polyNBi as a dielectric material. At the same time, the dielectric relaxation strength (Δε′) for polyNBi was approximately 8.0 at temperatures above T_g_ and at low frequencies.

**Figure 8:**
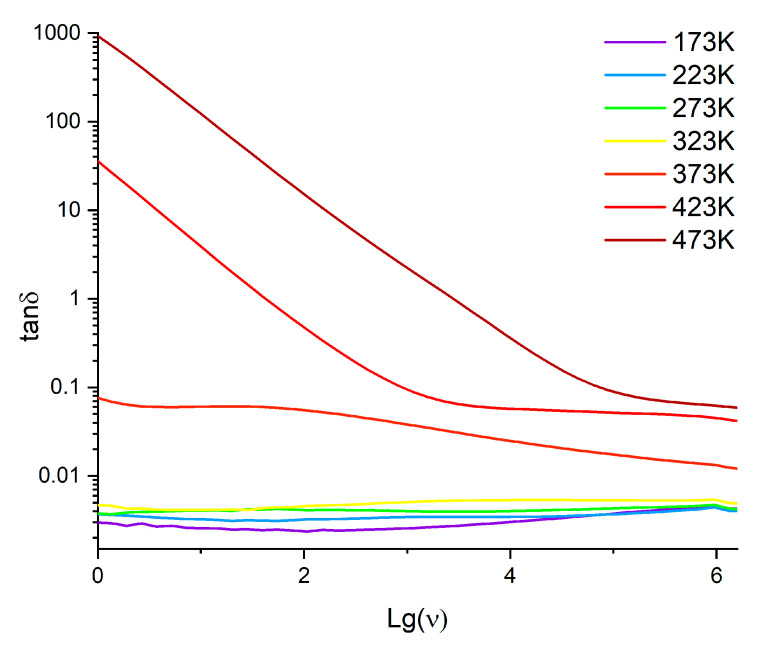
The frequency dependences of tanδ for polyNBi at various temperatures.

**Figure 9:**
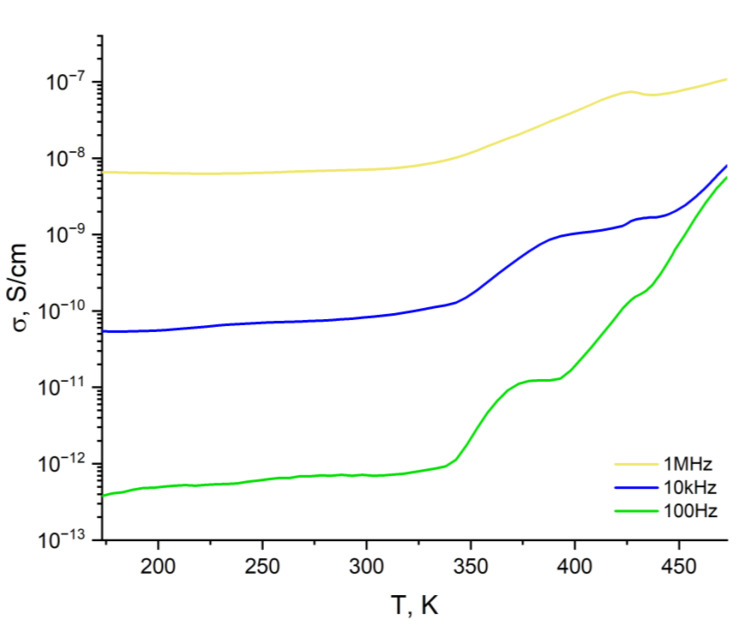
The temperature dependences of conductivity for polyNBi at different frequencies.

**Figure 10:**
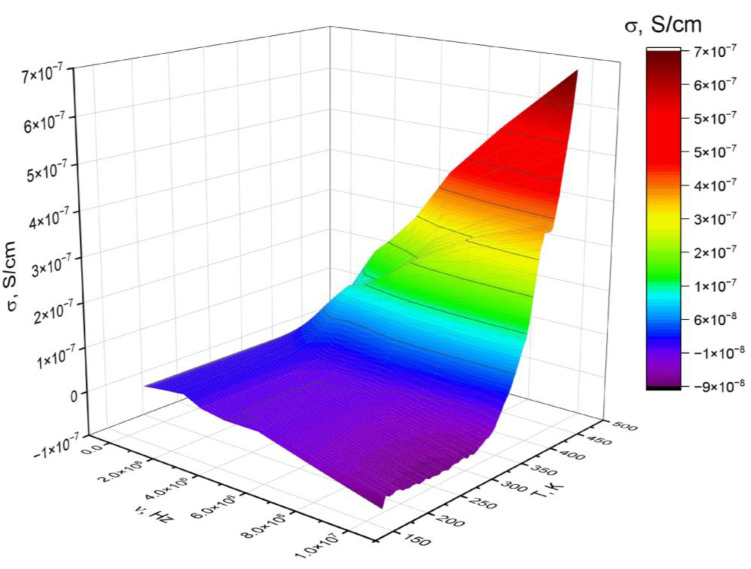
3D plot of conductivity versus temperature and frequency for polyNBi.

Dielectric loss (ε″) and tanδ are also very important properties of a dielectric material. Unexpectedly, polyNBi exhibited lower values of ε″ and tanδ than are typical for polar polymers (Table 3, Figure 8). Thus, the value of tanδ for polyNBi is between values for polymers with polar groups (tanδ~10^−2^) and for non-polar polymers (tanδ~10^−4^). This can be explained by the presence of large aromatic groups in side-chains of polyNBi. Furthermore, the value of tanδ for polyNBi did not depend on the frequency of the electric field and temperatures below T_g_. At the same time, at higher temperatures the value of tanδ was much higher, and it increased with temperature (Figure 8). Another interesting and important property of a dielectric material is its conductivity (σ) [41,42]. PolyNBi exhibited rather low values of conductivity at low and moderate temperatures and at low frequencies (below 10^−10^ S/cm, Figure 9 and Figure 10). The increase in temperature or frequency led to the fast growth of σ. Interestingly, at low temperatures (from 173 to 300 K), the conductivity increased linearly with temperature, while at higher temperatures there was a deviation of this linear dependence due to the appearance of flexibility polymer main and side-chains when temperatures were close to T_g_.

The review of the literature data describing the dielectric properties of metathesis polymers allows valuable comparisons to be made. Fang et al. synthesized metathesis polynorbornenes, bearing fluorinated eugenol groups. These polymers showed dielectric permittivity below 2.65 [9]. Metathesis polynorbornene with benzocyclobutene side-substituents exhibited the value of ε′ close to 2.60 [26]. The related ROMP polynorbornenes from N-substituted 5-norbornene-2,3-dicarboxyimides possessed values of ε′ in the range of 3–20 [43,44,45]. Therefore, polyNBi has one of the lowest values of ε′ among ROMP polynorbornenes. This result, in combination with the low values of dielectric loss and tanδ, allows us to consider polyNBi as a promising polymeric material for future and detail studies.

## 4. Conclusions

A new norbornene-type monomer with ester moiety (NBi) and its metathesis polymer (polyNBi) were synthesized from *exo*-5-norbornenecarboxylic acid and 1,1′-bi-2-naphthol. The designed monomer was prepared according to a two-step procedure in a good yield using conventional organic tools. The metathesis polymerization of this monomer over the 1-st generation Grubbs catalyst gave high-molecular-weight polymers with yields close to quantitative. The polymer exhibited good mechanical and film-forming properties. It was an amorphous high T_g_ polymer (161 °C). Despite the presence of bulky and rigid binol-groups in side chains, gas permeability of polyNBi was lower than that of metathesis polynorbornene without substituents (polyNB). Shown, unlike most, polyNBi is a classic low-permeable glassy polymer. This is unique to polynorbornenes with large side substituents, which tend to be highly permeable glasses. It was found that the selectivity of polyNBi was higher than that of polyNB. The high selectivity for the O_2_/N_2_ and the He/CH_4_ gas pairs for polyNBi also draws attention. At the same time, being less permeable than polyNB, polyNBi unexpectedly showed a lower value of dielectric permittivity (2.7 for polyNBi vs. 5.0 for polyNB). Therefore, the nature of the aryl group in the ester motif of side-chains can have a strong influence on dielectric permittivity through various kinds of interactions (dipole–dipole, π–π, etc.). Therefore, the molecular design of polynorbornenes has great potential to obtain polymers with desired properties in a wide range of required characteristics. Further tuning of the gas separation efficiency can be achieved by attaching an appropriate substituent to the ester and aryl group.

## Figures and Tables

**Table 1:** Metathesis polymerization of the ester of *exo*-5-norbornenecarboxylic acid and 1,1′-bi-2-naphthol ^a^.

Monomer/[Ru] Molar Ratio	C, M	Yield, %	M_w_·10^−3^	M_n_·10^−3^	M_w_/M_n_	T_g_, °C	[α]_D_,°^,b^
1000/1	0.5	97	786	236	3.3	161	−28
500/1	0.5	98	537	135	3.9		
1000/1	0.05	94	1500	438	3.4		

^a^-[Ru]–the 1-st generation Grubbs catalyst; solvent–1,2-dichloroethane; reaction temperature–25 °C; time of the polymerization–30 min. ^b^–the specific optical rotation was measured at 21 °C in chloroform (C = 1).

**Table 2:** WAXD data for polyNBi in comparison with unsubstituted metathesis polynorbornene (polyNB).

Polymer	*2θ*_1_, °	*2θ*_2_, °	*d*_1_, Å	*d*_2_, Å
polyNBi	11.8	19.7	7.5	4.5
polyNB [35]	18.1	-	4.9	-

**Table 3:** Permeability, diffusivity, and solubility coefficients of various gases in polyNBi and polyNB.

Polymer	Permeability (P), Barrer						
	He	H_2_	N_2_	O_2_	CO_2_	CH_4_	C_2_H_6_
polyNBi	8.4	8.1	0.10	0.61	2.67	0.13	0.046
polyNB [36]	19.4	13.0	0.75	2.6	13.9	1.9	-
PSF [37]	13	-	0.25	1.4	5.6	0.27	-
Diffusivity coefficients (D), D·10^8^, cm^2^/s							
polyNBi	550	140	0.87	2.9	0.82	0.22	0.011
Solubility coefficients (S), S·10^3^, cm^3^/(cm^3^·cm Hg)							
polyNBi	0.15	0.58	1.2	2.1	33	5.9	42

**Table 4:** Selectivities of separation for different pairs of gases for polyNBi and polyNB.

Permeability Selectivity						
Polymer	O_2_/N_2_	CO_2_/N_2_	CO_2_/CH_4_	He/N_2_	C_2_H_6_/CH_4_	He/CH_4_
polyNBi	6.1	26.7	20.5	84	0.35	64
polyNB [36]	3.5	18.5	7.3	30	-	10.2
PSF [37]	5.6	22	22	52	-	48
Diffusivity selectivity						
polyNBi	3.33	0.94	4.00	161	0.050	636
Solubility selectivity						
polyNBi	1.75	27.5	5.6	0.48	7.1	0.098

**Table 5:** Dielectric properties of some polynorbornenes (the values are given at 1 MHz and 298 K).

Polymer	ε′	ε″	tanδ	σ, S/cm
polyNBi	2.7	0.013	4.8·10^−3^	7.0·10^−9^
polyNB [40]	5.0	0.25	0.05	-
polyNBE [30]	3.3–8.9	-	-	-
polyNBDCAlk [29]	3.0–3.5	-	-	-

## Data Availability

NMR spectra of NBi and polyNBi are available free of charge as a file of Supplementary Materials.

## Supplementary Materials

The following supporting information can be downloaded at: https://www.mdpi.com/article/10.3390/polym14132697/s1, Figure S1. ^1^H NMR in CDCl_3_ (S)-2′-Methoxy-[1,1′-binaphthalen]-2-ol, Figure S2. ^1^H NMR spectrum of NBi in CDCl_3_, Figure S3. ^13^C NMR spectrum of NBi in CDCl_3_, Figure S4. DEPT-135 NMR spectrum of NBi in CDCl_3_, Figure S5. ^1^H,^1^H-COSY of NBi in CDCl_3_, Figure S6. ^1^H,^1^H-COSY (300 MHz) of NBi in CDCl_3_, Figure S7. ^1^H,^13^C-HMBC of NBi in CDCl_3_, Figure S8. ^13^C NMR spectrum of polyNBi in CDCl_3_.
