# Supplementary material for: Gas-Transport and the Dielectric Properties of Metathesis Polymer from the Ester of exo-5-Norbornenecarboxylic Acid and 1,1′-Bi-2-naphthol
Source: Polymers (Basel). 2022 Jun 30;14(13):2697. doi: 10.3390/polym14132697 (PMC9269233; doi:10.3390/polym14132697)
Supplement: Supplementary file 1 [file polymers-14-02697-s001.zip › polymers-1789329-supplementary.pdf]

Supplementary Material

Gas-Transport and the Dielectric Properties of Metathesis Polymer from the Ester of *exo*-5-norbornenecarboxylic Acid and 1,1′-bi-2-naphthol

Ivan V. Nazarov,[1] Danila S. Bakhtin,[1] Ilya V. Gorlov,[1,2] Konstantin V. Potapov,[3] Ilya L. Borisov,[1] Ivan V. Lounev,[4] Igor S. Makarov,[1] Alexey V. Volkov,[1] Eugene Sh. Finkelshtein[1] and Maxim V. Bermeshev[1,]*

[1]A.V. Topchiev Institute of Petrochemical Synthesis of Russian Academy of Sciences, 29 Leninsky prospekt, 119991 Moscow, Russia

[2]The Moscow State University, Faculty of Fundamental Physical and Chemical Engineering, 1 Leninskie Gory, 119991 Moscow, Russia

[3]N.D. Zelinsky Institute of Organic Chemistry of the Russian Academy of Sciences, 47 Leninsky Prospect, 119991 Moscow, Russia

[4]Institute of Physics, Kazan Federal University, 18 Kremlyovskaya street, 420008 Kazan, Russia

* Correspondence: bmv@ips.ac.ru (M.V.B.)

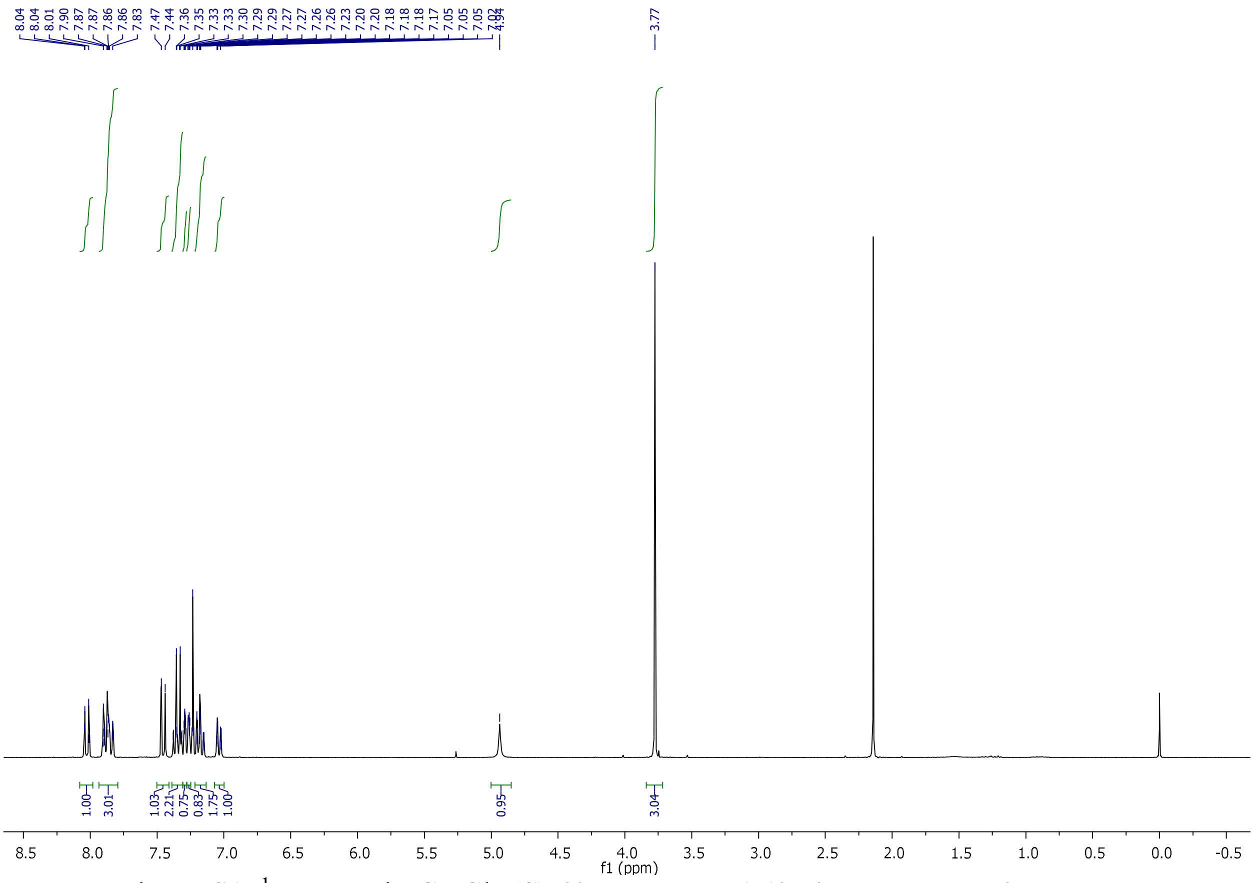

Figure S1. ^{1}H NMR in $CDCl_3$ **(S)-2'-Methoxy-[1,1'-binaphthalen]-2-ol**.

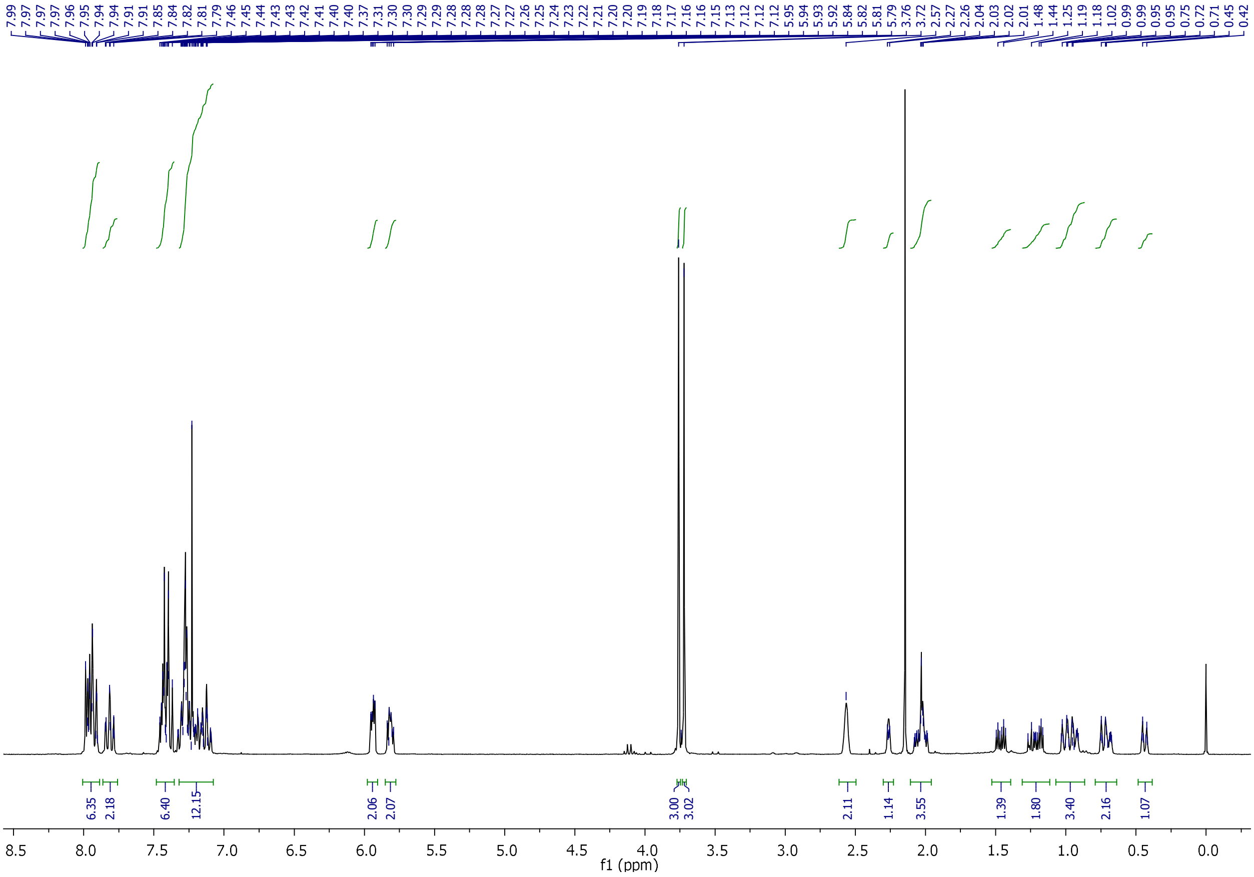

Figure S2. ^{1}H NMR spectrum of **NBi** in $CDCl_3$.

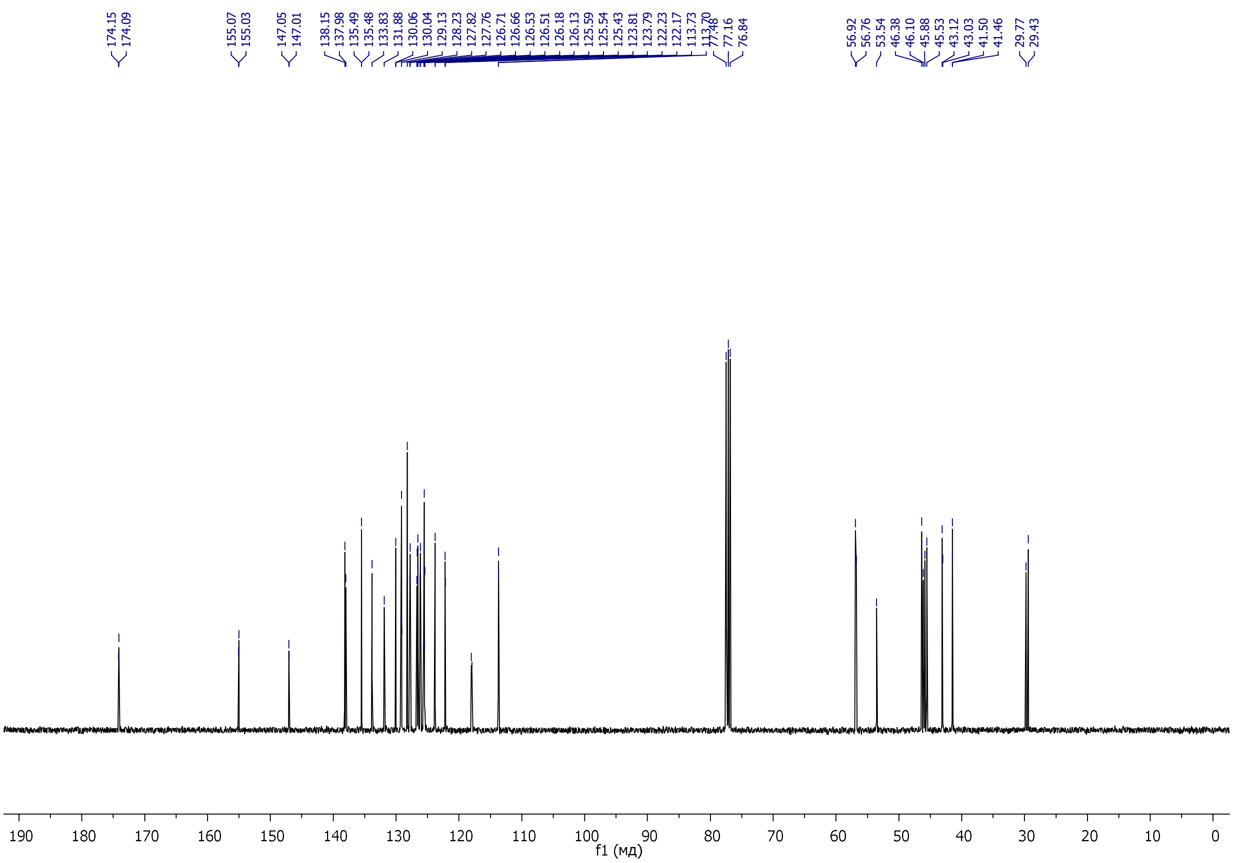

Figure S3. ^{13}C NMR spectrum of **NBi** in $CDCl_3$.

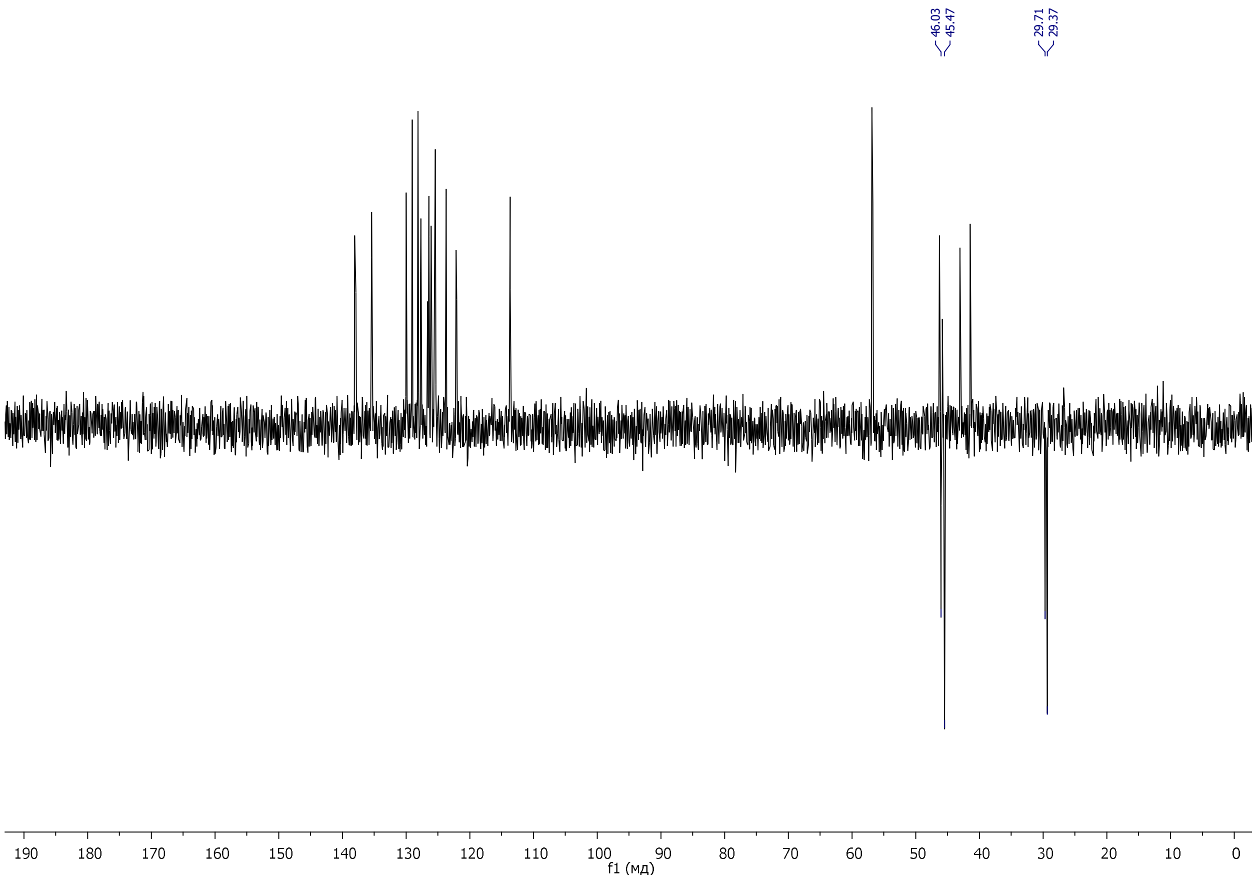

Figure S4. DEPT-135 NMR spectrum of **NBi** in $CDCl_3$.

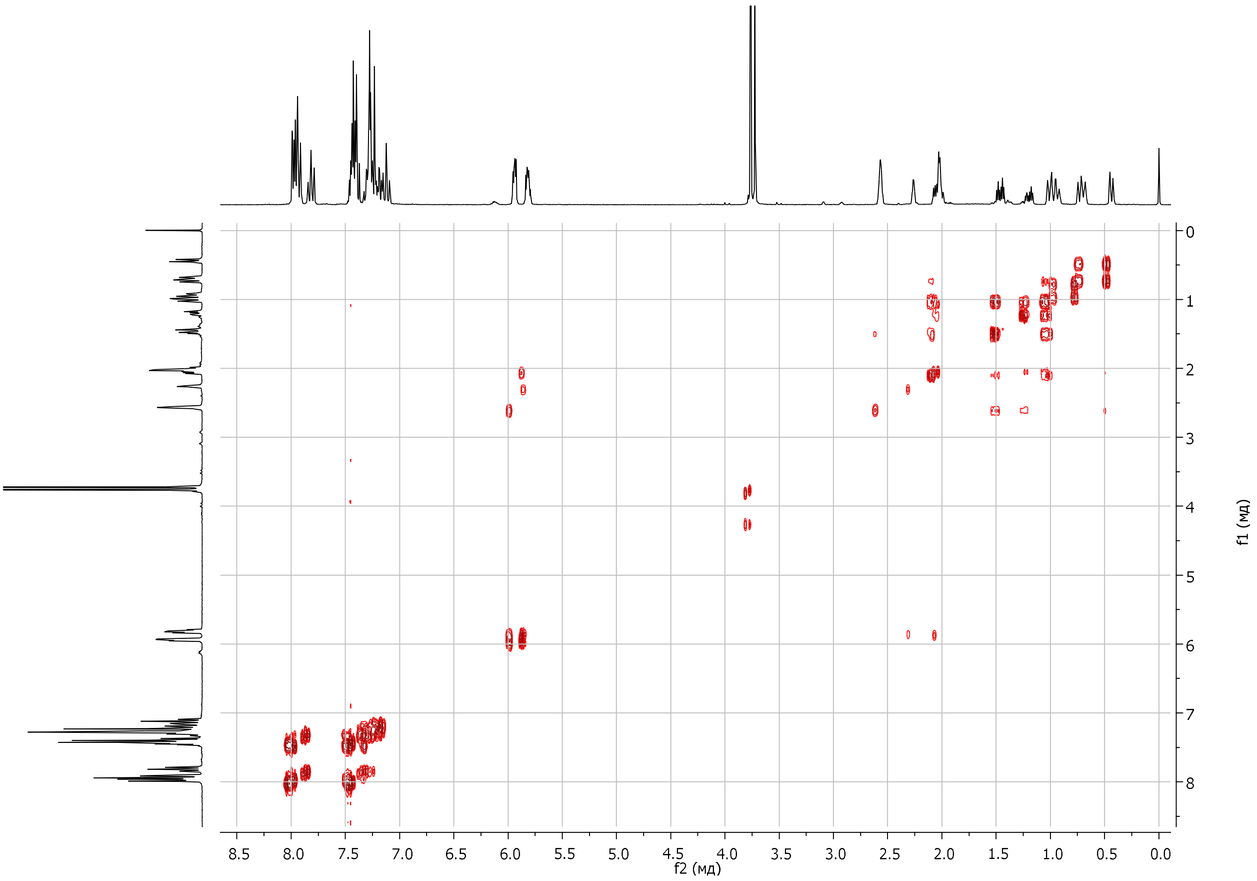

Figure S5. $^{1}H,^{1}H$-COSY of **NBi** in $CDCl_3$.

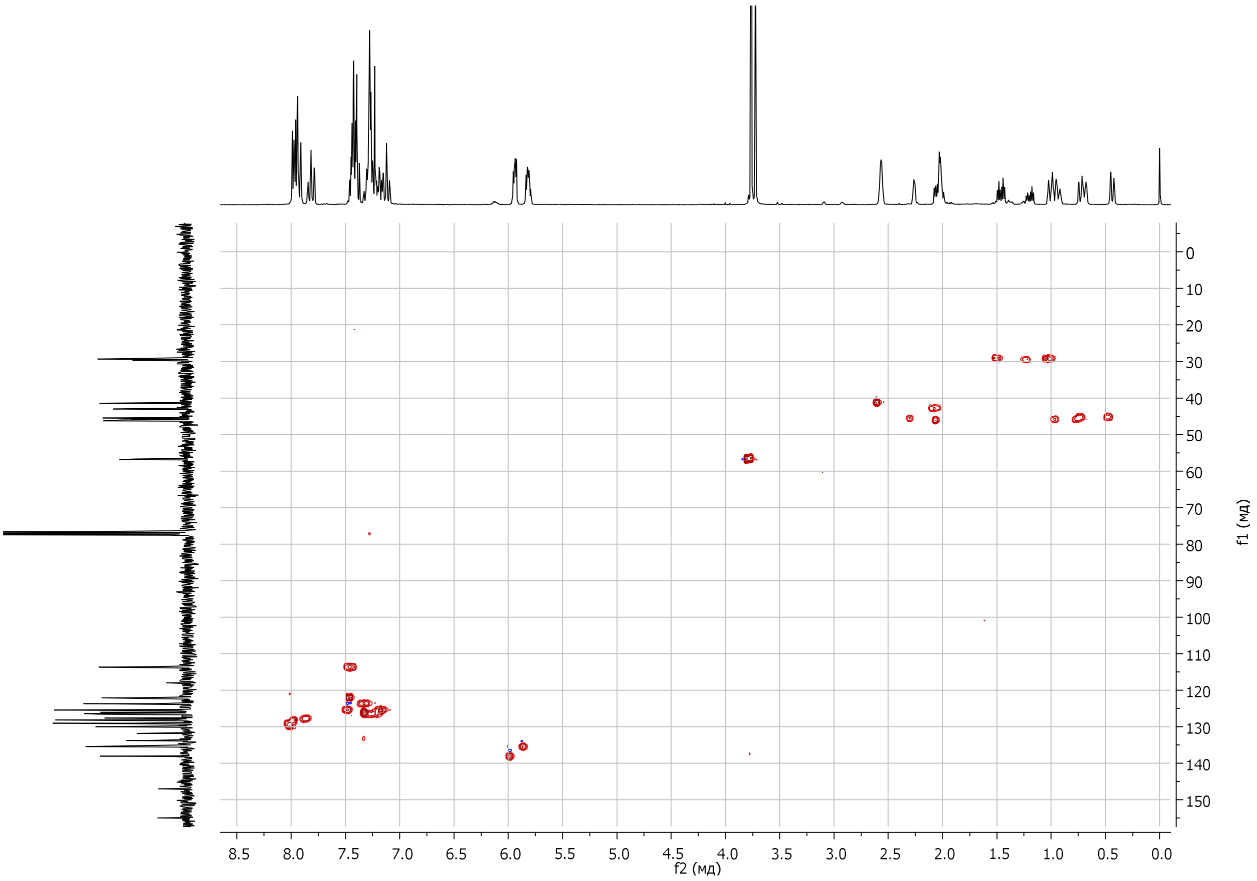

Figure S6. ^{1}H,^{1}H-COSY (300 MHz) of **NBi** in $CDCl_3$.

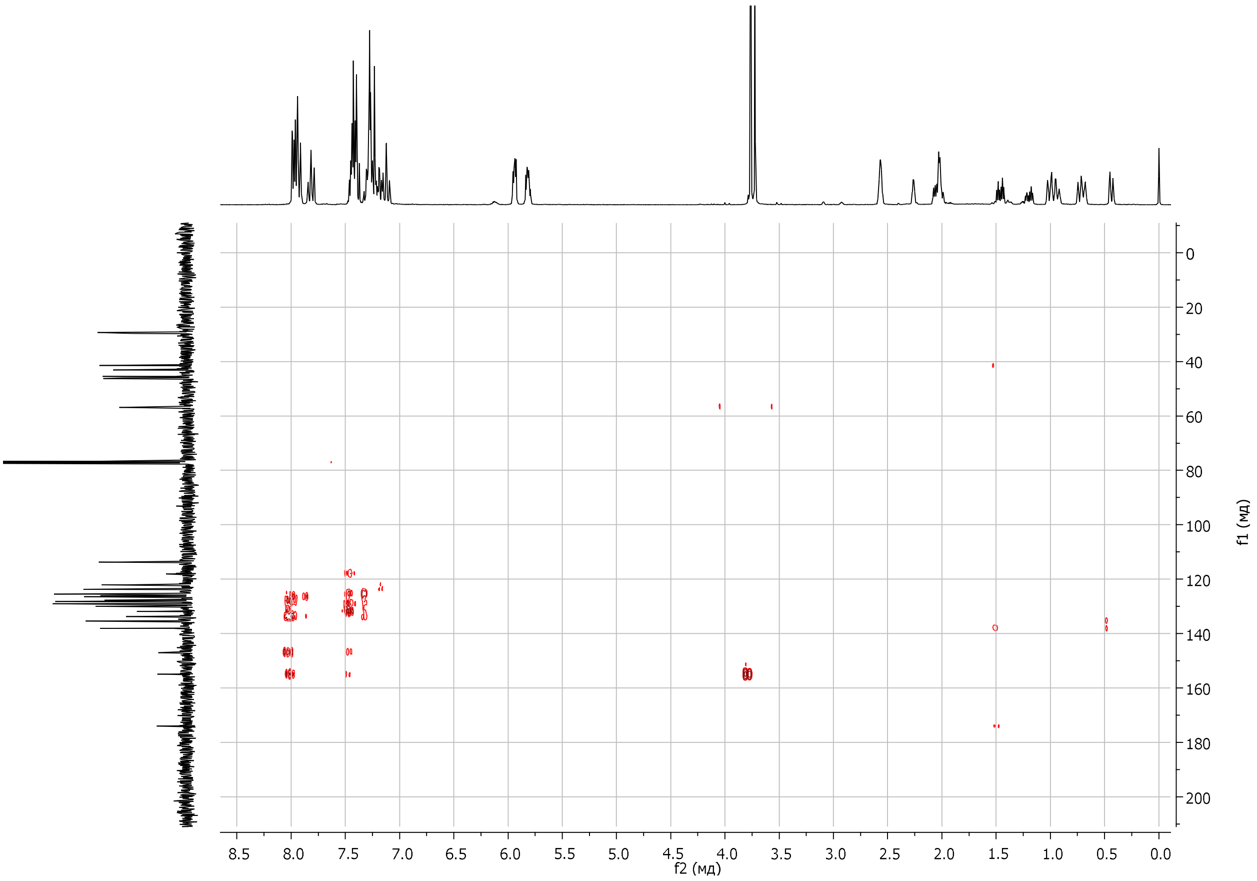

Figure S7. $^{1}H,^{13}C$-HMBC of **NBi** in $CDCl_3$.

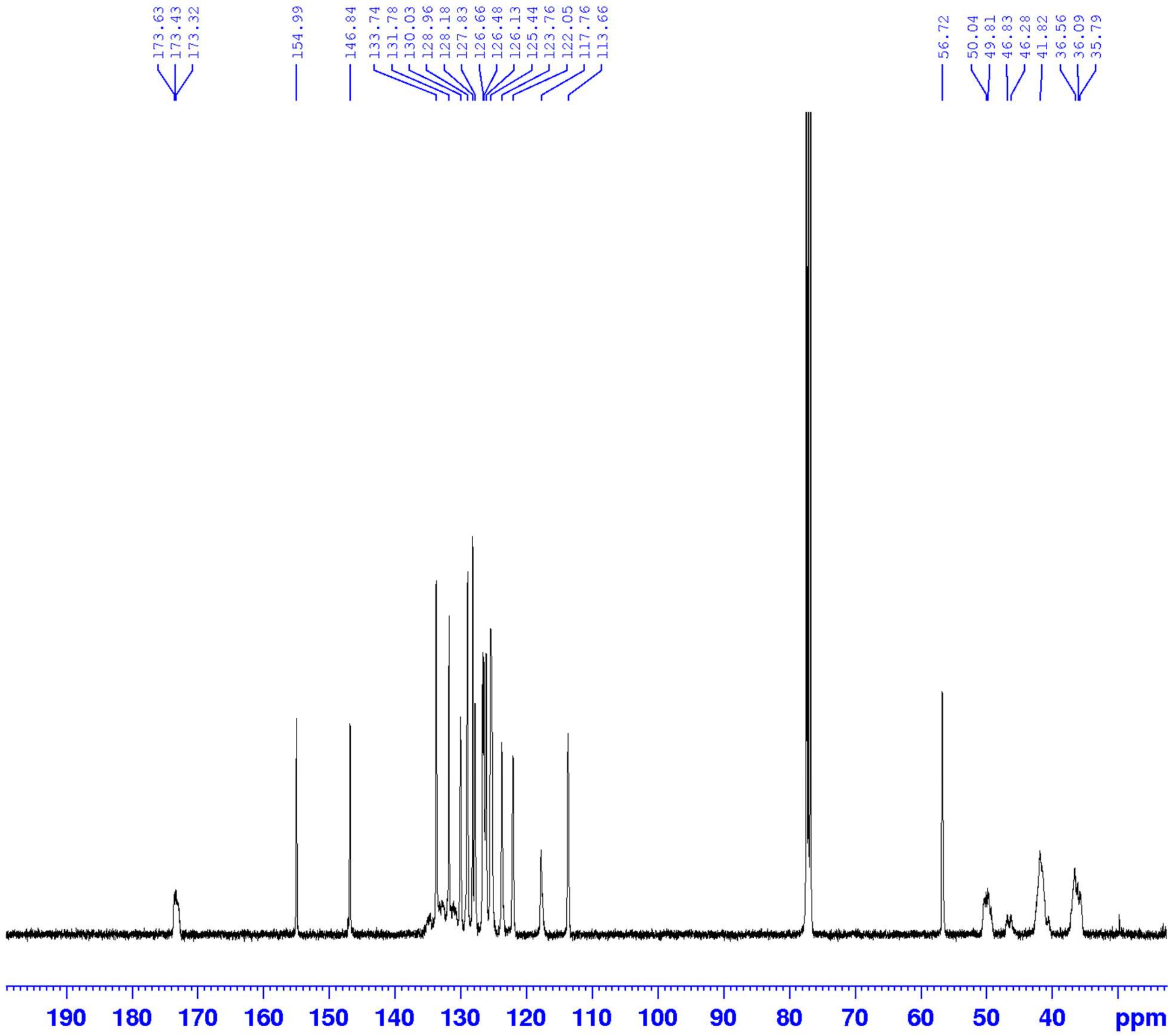

Figure S8. ^{13}C NMR spectrum of **polyNBi** in $CDCl_3$.
